# Sellar and suprasellar arachnoid cyst

**DOI:** 10.31744/einstein_journal/2019AI4269

**Published:** 2019-01-28

**Authors:** João Mangussi-Gomes, André Felix Gentil, Renée Zon Filippi, Rafael Almeida Momesso, Benjamin Wolf Handfas, João Radvany, Leonardo Balsalobre, Aldo Cassol Stamm

**Affiliations:** 1 Complexo Hospitalar Edmundo Vasconcelos, São Paulo, SP, Brazil.; 2 Hospital Israelita Albert Einstein, São Paulo, SP, Brazil.

A 36-year-old woman admitted with a 2-week history of headaches and blurred vision. Her medical history was positive for irregular menses and hypothyroidism. Visual field tests revealed defects in the upper quadrants bilaterally and blood tests indicated slightly elevated prolactin levels (24.4; range 4.8 to 23.3), reduced morning cortisol (3.8; range 4 to 22), and reduced growth hormone levels (<0.05; range 0.13 to 9.88).

**Figure 1:**
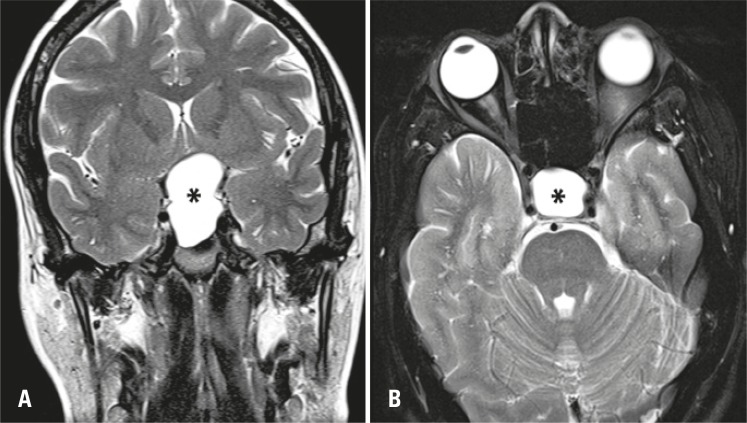
T2-weighted magnetic resonance images. (A) Coronal view revealing a large hyperintense and well-delineated sellar lesion (asterisk) with significant suprasellar extension. (B) Axial view. The homogeneous lesion (asterisk) occupies the whole sella turcica

**Figure 2:**
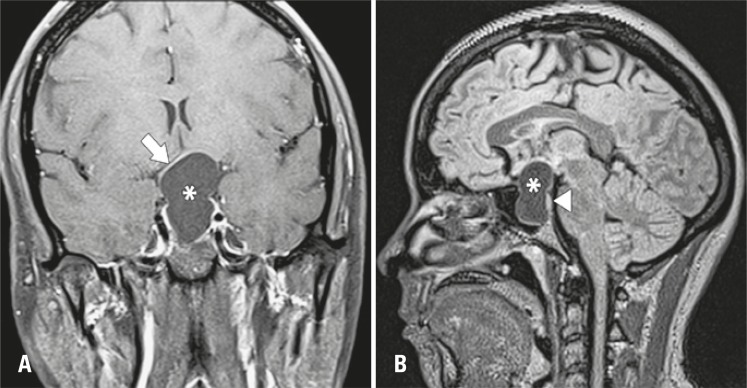
T1-weighted magnetic resonance images. (A) Coronal image after gadolinium administration. The lesion (asterisk) shows thin walls with slight rim enhancement (arrow). (B) Sagittal FLAIR magnetic resonance image. Note the sellar and suprasellar extension of the lesion and the pituitary gland flattened against the *dorsum sellae* (arrowhead)

Magnetic resonance imaging identified a well-delineated, homogeneous, cystic sellar lesion with suprasellar extension and thin walls. The pituitary gland and stalk appeared to be stretched over the cyst boundaries and compressed against the *dorsum sellae* . No calcifications or solid areas were identified ( Figures 1 and 2 ).

**Figure 3:**
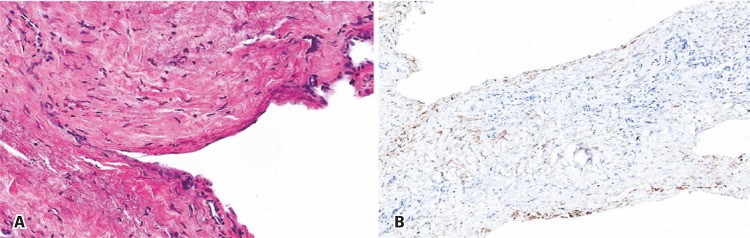
Histopathological and immunohistochemical analysis of the surgical specimen. (A) Histopathological image of the surgical specimen (hematoxylin & eosin, 200x). Note the fibrous matrix lined by the flattened arachnoidal cells, and the absence of ciliated or squamous cells. (B) Immunohistochemical analysis was positive for epithelial membrane antigen, corroborating the diagnosis of arachnoid cyst

The patient underwent endoscopic endonasal surgery, and the lesion was decompressed. She had good evolution after the surgery, and pathological examination of the walls confirmed the diagnosis of an arachnoid cyst ( Figure 3 ).

The sellar region can be affected by a variety of non-pituitary cystic lesions, including craniopharyngiomas, Rathke’s cleft cysts, and arachnoid cyst.^(^ ^1^ ^)^ Such lesions represent only 5.5% of all sellar lesions, with arachnoid cyst comprising up to 20% of them.^(^ ^2^ ^,^ ^3^ ^)^ Sellar arachnoid cyst is therefore rare, representing only 0.6 to 0.8% of all sellar lesions.^(^ ^2^ ^,^ ^4^ ^)^

Radiological differentiation between craniopharyngiomas, Rathke’s cleft cysts, and arachnoid cyst is key for preoperative planning and prognosis, but sometimes it can be challenging.^(^ ^1^ ^,^ ^5^ ^)^

On magnetic resonance imaging, craniopharyngiomas appears as a heterogeneous, mixed (solid and cystic), and often calcified lesion. In contrast, Rathke’s cleft cysts and arachnoid cyst show smooth-contours and homogeneous lesions.^(^ ^2^ ^,^ ^5^ ^)^ Rathke’s cleft cysts typically has a midline location, and originates between the anterior and posterior pituitary, with MR signal intensity depending on its proteinaceous/mucinous content, and most Rathke’s cleft cysts will depict an intracystic solid nodule. Rathke’s cleft cysts does not enhance, but might exhibit an enhancing rim of compressed pituitary tissue surrounding the cyst (claw sign).^(^ ^6^ ^)^ Similarly, arachnoid cyst shows no contrast enhancement and the pituitary gland can be seen flattened against the sellar walls.^(^ ^7^ ^)^ Arachnoid cyst contains exclusively cerebrospinal fluid, being therefore the most homogeneous and less variable lesion among all of them, as in the present case.^(^ ^1^ ^,^ ^2^ ^)^
